# Epoxy Resins for Flooring Applications, an Optimal Host for Recycling Deactivated Cement Asbestos

**DOI:** 10.3390/polym15061410

**Published:** 2023-03-12

**Authors:** Fabrizio Campanale, Fabrizio Vergani, Narcisa Mihaela Marian, Cecilia Viti, Alberto Bianchi, Silvia Ferrario, Michele Mauri, Giancarlo Capitani

**Affiliations:** 1 Department of Earth and Environmental Sciences (DISAT), University of Milano-Bicocca, Piazza della Scienza 4, 20126 Milano, Italy; fabrizio.campanale@unimib.it (F.C.); fabrizio.vergani@unimib.it (F.V.); 2 Department of Physical Science, Earth, and Environment (DSFTA), University of Siena, Via Laterina 8, 53100 Siena, Italy; narcisamihaela.ma@student.unisi.it (N.M.M.); cecilia.viti@unisi.it (C.V.); 3 Graftonica Srl, Via Martiri Triestini 7, 20148 Milan, Italy; alberto.bianchi@graftonica.it (A.B.); silvia.ferrario@graftonica.it (S.F.); 4 Department of Materials Science, University of Milano-Bicocca, Via Roberto Cozzi 55, 20125 Milan, Italy; michele.mauri@unimib.it

**Keywords:** recycling, epoxy resin, inorganic filler, deactivated asbestos, flooring applications

## Abstract

Cement asbestos slates, commonly known as Eternit^®^ and still abundant in private and public buildings, were deactivated through a thermal process. The resulting deactivated cement asbestos powder (DCAP), a mixture of Ca-Mg-Al silicates and glass, was compounded with Pavatekno Gold 200 (PT) and Pavafloor H200/E (PF), two different epoxy resins (bisphenol A epichlorohydrin) for flooring applications. The addition of the DCAP filler to the PF samples causes a slight but acceptable decrease in the relevant mechanical properties (compressive, tensile, and flexural strengths) upon increasing DCAP content. The addition of the DCAP filler to pure epoxy (PT resin) causes a slight decrease in the tensile and flexural strengths with increasing DCAP content, while the compressive strength is almost unaffected, and the Shore hardness increases. The main mechanical properties of the PT samples are significantly better than those of the filler-bearing sample of normal production. Overall, these results suggest that DCAP can be advantageously used as filler in addition to, or in substitution for, commercial barite. In particular, the sample with 20 wt% of DCAP is the best performing in terms of compressive, tensile, and flexural strengths, whereas the sample with 30 wt% of DCAP shows the highest Shore hardness, which is an important property to be considered in flooring applications.

## 1. Introduction

The massive increase in harmful by-products of industrial activities in recent decades has triggered an equally massive impulse for their treatment. The minimum goal of removing risks for the environment and health has been later complemented by the aim of reinserting the treated materials within the economic cycle.

The goal of this work is to use the powder obtained by the deactivation of cement asbestos slates (CAS) through thermal treatments [1,2] as a new non-conventional filler for polymer composites. Asbestos is a term used to indicate the fibrous minerals belonging to the serpentine and amphibole groups [3] employed as raw materials worldwide since the middle of the past century. Although asbestos has been classified as a carcinogen and banned in almost all countries since the 1990s, asbestos-containing materials (ACM) are still abundant in many buildings and represent a hazard for human health. The current strategies adopted by law aiming at mitigating the asbestos hazard are (i) confinement, i.e., insulating the ACM from the exterior through a physical barrier; (ii) encapsulation, i.e., stabilizing the asbestos fibres with special resins such as to prevent their release into the environment; and (iii) landfilling, i.e., removal and disposal of the ACM in controlled landfills. None of these solutions is ideal because the risk of fibre release in the environment remains high and a potential secondary raw material is lost forever. The European Parliament resolution of 14 March 2013 on asbestos-related occupational health threats and prospects for abolishing all existing asbestos (2012/2065(INI)) instead suggests processing with an energy-sustainable detoxification treatment and reusing the deactivated material. If realized, it would protect the natural environment from pollution, minimise the depletion of non-renewable resources, and protect human health from hazards [1,2,4].

In recent years, a number of recycling routes of thermally treated CAS have been investigated, providing satisfactory results (for a review, see [5]). Among them, recycling in ceramics is by far the most exploited and one of the most promising (e.g., [4,6,7]). However, the amount of product that can be employed in ceramics without compromising the workability of the slurry is quite modest (≤5 wt% [4,6]).

Recycling or upcycling of an inorganic powder can often be achieved by its application as a filler in polymers. In this case, a much larger amount of recycled material can be employed (up to 60 wt%, e.g., [8,9,10,11,12,13]). Fillers are solid-form additives basically different from the polymeric matrix in terms of composition and structure. They are commonly added for cost reduction, but the addition of mineral fillers into a polymer can improve the various properties including thermal and mechanical properties, creep resistance stiffness, shrinkage, and heat deflection temperature. Composite materials in which the components are synergistically combined to address the required application [14] are gaining acceptance in various types of engineering applications. However, the presence of the mineral filler sometimes deteriorates toughness and strength [15].

The physical and chemical properties of the filler are very crucial because they determine the material’s performance [16]. Notably, the effect of inorganic fillers on the composite’s mechanical properties can be influenced by various factors, such as the composite’s shape, particle size, aggregate size, surface morphology, and general matrix properties [17,18,19]. Aggregation also depends on the chemical nature of the filler surface and its interactions with the polymer matrix: in some cases, a surface treatment [20] or even the grafting of polymer chains to match the matrix [21] is needed to achieve good dispersion. Generally, the polymer industry demands fillers with very fine particle size, preferably below 10 μm, with narrow particle-size distribution and particle shapes according to the function of the filler in the organic matrix. Specifically, an elongated and flaky particle shape is essential for tensile strength, whereas a spherical and cubical particle shape improves impact strength.

Commonly used mineral fillers in the polymeric industry are talc, limestone, silica, clay, mica, wollastonite, and barite. Sheet-like platy fillers, such as talc, mica, and kaolin, have been reported to enhance rigidity [22]. In addition, mica has excellent thermal insulating properties that reduce flammability [23,24]. Silica is notable for its extremely low thermal expansion coefficient. Limestone (CaCO_3_) is regarded as an inexpensive mineral filler that can be utilized at high filler loadings and improve the flexural modulus. It also exhibits an excellent surface finish, as well as viscosity control [25].

In terms of production volume and consumption, epoxy resins occupy an important place in the polymer industry. A variety of filler–epoxy composites have been tested so far, with the aim of either reducing the cost of finished products by reducing the cost of expensive binders or to give to the composite various functional properties. Among the low-cost, micron-sized mineral fillers so far investigated, there are fly ash [11,26], brick dust [9], granite dust [8,27], chromite [10], marble dust [28,29], ochre [12], and diorite dust [13].

In the present paper, we employed thermally deactivated cement asbestos powder (DCAP) as a filler in the epoxy resin used for flooring applications, without further surface treatment. These applications were selected because they require good mechanical properties and are usually fulfilled by long-lasting thermoset materials. The results show that, for some formulations, the mechanical properties of DCAP-bearing composites are superior compared to those of normal production, suggesting a promising recycling route for the DCAP that matches the rising demand for more efficient materials in terms of low costs, sustainability, and improved mechanical behaviour and processing.

## 2. Materials and Methods

### 2.1. Materials

DCAP was mixed as inorganic filler with two different bisphenol A (BPA) epichlorohydrin (ECH)-based epoxy resins. The resins are commercially known as Pavafloor H200/E (henceforth Pavafloor or PF) and Pavatekno Gold 200 (henceforth Pavatekno or PT), both produced by Pava Resine Srl and typically used for flooring in civil and industrial buildings. The PT is a filler-free BPA ECH resin, while PF is a solvent-free pigmented epoxy formulation with a pre-existing inorganic filler of barite included in the resin. PF is also a BPA ECH resin, but according to datasheet, it also contains 10 wt% polypropylene glycol (PPG)-epichlorhydrin copolymer and 4 wt% di(propylene glycol) dibenzoate. Both resins were combined with the hardener, composed of a mixture of benzyl alcohol, isophorone diamine (IPDA), and an oligomer of IPDA with epichlorohydrin and bisphenol.

The samples were prepared by adding 10%, 20%, and 30% wt% of DCAP to the PT resin, and 2%, 5%, and 10 wt% to the PF resin, according to Table 1. The reduced loading of DCAP in PF resin (compared to PT) is due to the presence of pre-existing barite in the mixture (38 wt%, see ahead). We also prepared reference samples with 0 wt% of DCAP for comparison. Before the sample preparation, the DCAP was dried in oven for 24 h at 100 °C and sieved to remove the >80 µm fraction.

Due to the lower viscosity of the hardener compared to the resins, we first mixed the DCAP with the hardener using a laboratory dissolver mixer for 5 min. We then added the resin and repeated the mixing with the dissolver mixer for another 5 min. The mixture was then left in a vacuum chamber for about 5 min to remove the entrapped air, filled into silicon moulds (with suitable shapes for the mechanical tests, see Section 2.2) and finally cured for 14 days at ~20 °C.

**Figure 1:**
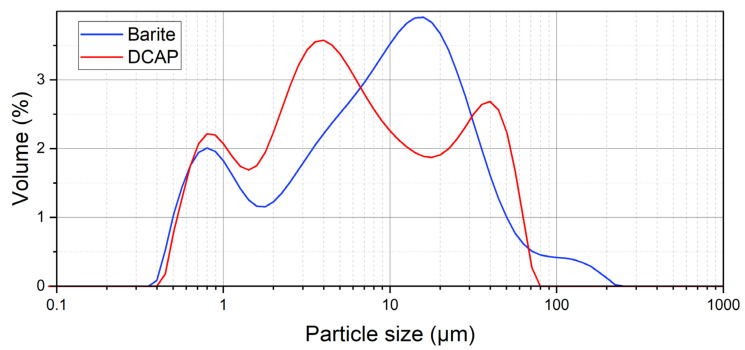
DLS grain size distribution of DCAP [2] and barite (this work).

DCAP consists of a mixture of glass and Ca-Mg-Al silicates typical of cements, as a result of in-air heat treatment at ~1100 °C of CAS and subsequent 10 min milling in ball mill [1,2,4]. The DCAP used here is the same exhaustively investigated by Vergani et al. [2] through a combination of state-of-the-art techniques, including X-ray powder diffraction (XRPD), X-ray fluorescence spectrometry (XRF), dynamic laser scattering (DLS), scanning (SEM), and transmission electron microscope (TEM). For reader convenience, we summarize in Table 2 the main properties of DCAP, while in Figure 1 we compare the grain size distribution of DCAP with that of barite originally present in PF samples, obtained by DLS (more details in [2]). The barite content of the PF0 sample was obtained through a thermal gravimetric analysis (TGA), resulting in 38 wt% (Figure S1 in the supporting material).

### 2.2. Mechanical Properties Determination (UTM) and Hardness

The stress/strain behaviour of the samples, including compressive strength, uniaxial tensile strength, and flexural strength tests, were obtained using a Zwick-Roell Retroline universal testing machine (UTM), equipped with a 5 kN load cell and several appropriate tools. Each test was carried out with at least 4 measurements and the results were averaged, as reported in Table 3 and Table 4.

Compression test was performed on a ~5 × 12.5 mm resin cylinder at a rate of 1.3 mm/min. Tensile test was obtained by stretching a dumbbell-shaped specimen of resin with dimension according to ISO 527 type 1, and a rate of 1 mm/min. Flexural test was obtained by bending a rectangular prism of resin 2 × 2 × 30 mm at a rate of 1 mm/min.

Hardness test was carried out using a Vulcanoline digital Shore durometer, model VLDSD6610D, with a conical R0.1 indenter, DIN 53505 and ASTM D2240 type D scale, and a resulting force of 44.48 N. All data, 10 measurements for each sample, are reported in Table 5.

### 2.3. Thermal Properties Determination (DSC)

Glass transition temperature (T_g_) was determined by differential scanning calorimetry (DSC) with a Mettler-Toledo DSC1 Star System under a nitrogen atmosphere. The instrument was calibrated using Indium melting temperature and enthalpy, and corrected for empty cell baseline. About 10–15 mg in mass was extracted from a thin film of each sample by cutting a disk of ~3 mm in diameter and encapsulated into 40 μL Al pan. The experiments were conducted by performing two heating and one cooling ramp, between −20 °C and 160 °C, at 20 °C/min. The T_g_ values calculated with the midpoint method for each sample are reported in Table 6, while raw DSC curves are in Figures S2 and S3.

### 2.4. Composite Morphology

Secondary electron (SE) images and energy dispersive X-ray (EDX) analyses were acquired at the Platform of Microscopy of the University of Milano-Bicocca with a Tescan VEGA TS 5136XM and a Zeiss Gemini 500 scanning electron microscopes (SEMs). SE images were acquired on the breaking surfaces after performing tensile and flexural tests to gain insights into the fracture mechanism. To avoid electrostatic charging, the samples were coated with a thin (~20 nm) graphite film.

## 3. Results and Discussion

### 3.1. Mechanical Properties 

The influence of fillers on the mechanical properties of a composite is determined by their size, shape, and nature of the interaction with the matrix [30]. The deactivation process of CAS presented in Marian and Vergani [1,2] radically changes the mineralogy by transforming the asbestos fibres into a mixture of glass and micro-to-nano particles of Ca-Mg-Al silicates. In principle, the mechanical properties of composites made with this new material are not related with asbestos and should be tested extensively.

**Figure 2:**
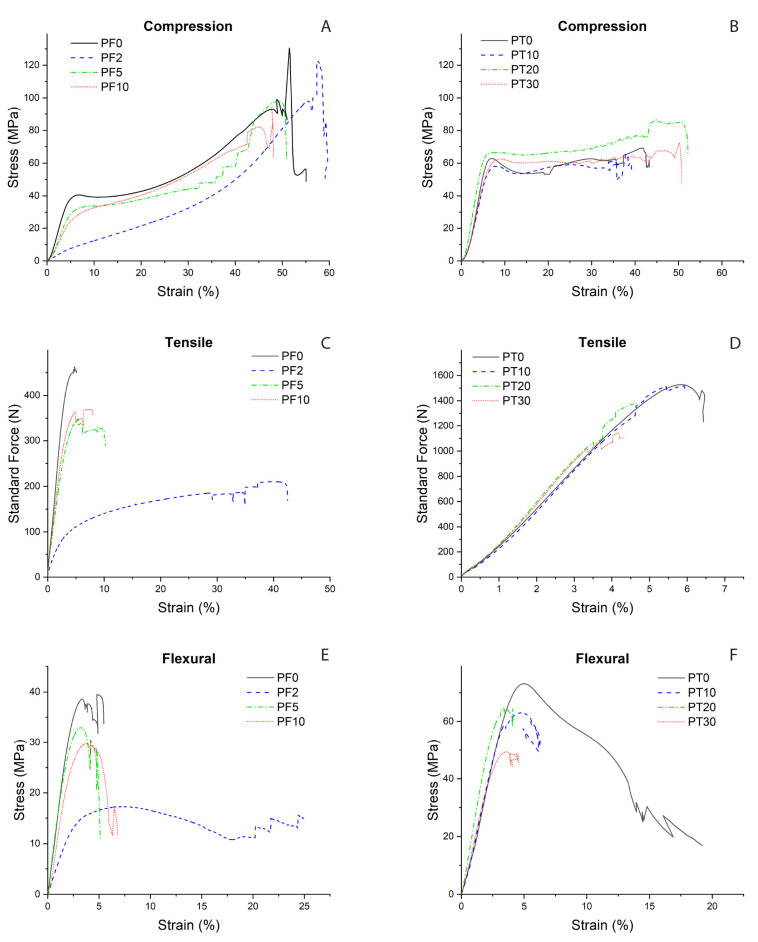
Effects of the DCAP filler amount on the compressive strength (**A**,**B**), tensile strengths (**C**,**D**), and flexural strengths (**E**,**F**) for the PF (**A**,**C**,**E**) and PT (**B**,**D**,**F**) composite samples.

**Figure 3:**
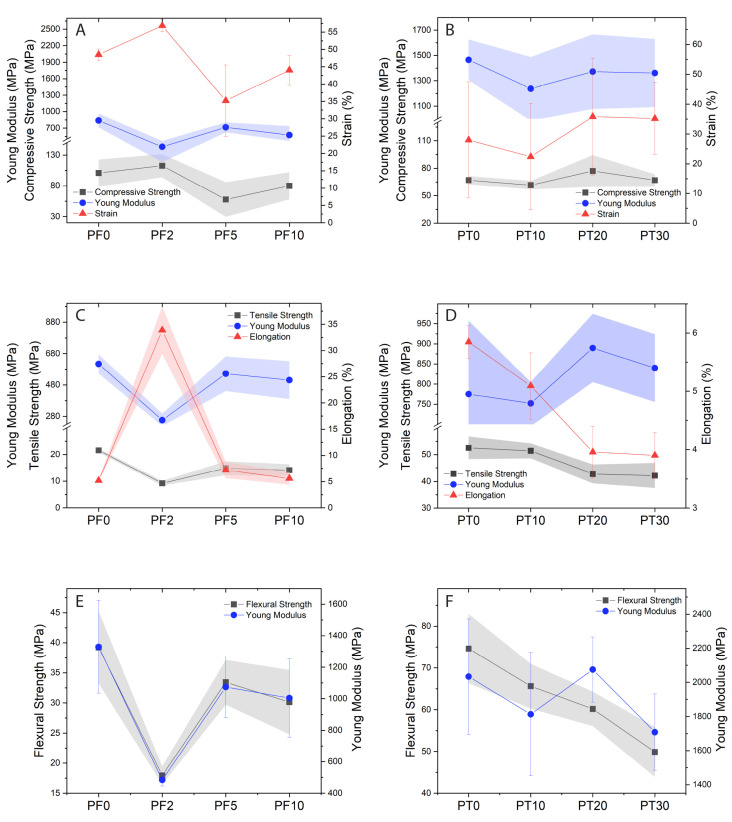
Variation of strength (compressive, tensile, and flexural), Young’s modulus, and deformation (strain and elongation) for the PF (**A**,**C**,**E**) and PT (**B**,**D**,**F**) composite samples.

Figure 2 and Figure 3 show the variation occurring in the compressive, uniaxial tensile, and flexural strengths of the composites as functions of the DCAP filler content. 

The DCAP-bearing PF samples show quite similar compressive stress–strain curves compared to the PF0, apart from sample PF2, which shows higher strain values under same stress conditions (Figure 2A and Figure 3A), i.e., lower elasticity and higher plastic deformation. In fact, PF2 displays an increase in mean strain value (up to ~60%) compared to other samples (35–50%), followed by a decrease in Young modulus (~143 MPa vs. 574–840 MPa in the other samples; Figure 3A, Table 3). Excluding the PF2 sample, we observe a slight decrease in compressive strength, Young modulus, and strain going from sample PF0 to samples PF5 and PF10. 

The anomalous behaviour of the PF2 sample can also be tracked on the tensile and flexural tests, where the PF2 sample shows a completely different stress/force–strain curve (Figure 2C,E). In comparison to the PF0 sample, the mean tensile strength and Young modulus of the PF2 sample are much lower (~9 vs. 21.6 MPa and 256 vs. 614 MPa, respectively), whereas the elongation is much higher (~34 vs. ~5%) (Figure 3C, Table 3). Similarly, the flexural strength and Young modulus of the PF2 sample are, respectively 18 MPa and ~489 MPa vs. ~39 MPa and ~1329 MPa for the PF0 sample (Figure 3E, Table 3). As for the compression test, in both tensile and flexural tests we observe a slight decrease in tensile/flexural strength and Young modulus going from sample PF0 to samples PF5 and PF10. 

Therefore, the increase in DCAP in the PF samples tends to slightly worsen the mechanical properties. However, their decrease can be considered acceptable especially considering the large amount of inorganic filler (DCAP + barite) mixed with the resin. A decrease in the properties of polymers are also not surprising when they are mixed with particles with low specific surface area, as is the case of DCAP − being composed by particles/aggregates up to 80 µm (Figure 1). Generally, an improvement of the mechanical properties of resins is recorded in the presence of small particles, while too large particles (~100 µm) promote failures at the filler–resin contact and wear reduction [31,32]. The potential economic benefit arising from the use of minor quantity of resin per unit volume must be also considered, due to the addition of lower-cost inorganic material in the mixture.

The anomalous behaviour of PF2 was initially considered to be due to a problem during the sample preparation. Then, we reconsidered this hypothesis after the preparation of a new sample that showed similar results. It seems that the addition of 2 wt% of DCAP to a resin already containing 38 wt% of barite builds up a critical filler concentration that dramatically affects the mechanical properties of the resin. A similar phenomenon was observed by [33] on talc-bearing poly(lactic acid), describing a decrease in viscosity for low talc concentration (1 wt%), i.e., “lubricant effect”, while showing an increase in viscosity with talc concentrations up to 7 wt%. However, the inorganic loads involved in the two compared cases are significantly different (1 wt% vs. 40 wt%), therefore excluding that a lubricant effect could be the cause of the observed behaviour, which remains unexplained.

The mechanical tests on PT samples generally show more consistent stress–strain curves as function of increasing DCAP fraction (Figure 2), albeit with a large variability of Young modulus and deformation values in each measurement (Figure 3). In the compressive test, the DCAP-bearing PT samples show quite similar stress–strain curves and are comparable with PT0 (Figure 2B). The PT20 sample, however, displays slightly higher stiffness, i.e., higher stress values are required to obtain the same strain (Figure 2B). We observe an increase in the compressive strength from 67 MPa (PT0) to ~83.5 MPa (PT20), and then a return to a mean value of 67 MPa for sample PT30 (Table 4). Further, we observe a slight decrease in the Young modulus from ~1465 MPa (PT0) to ~1360 MPa (PT30), with a consequent increase in the mean deformation value from 28% to ~35% (Figure 3B, Table 4). 

In the tensile test, PT samples retain the same stress–strain trends regarding the DCAP content (Figure 2D), but show a progressive reduction in both mean values of tensile strength (from ~52 to ~42 MPa) and elongation (from 5.8 to 3.9%) and an increase in the mean value of Young modulus (from ~775 to ~840 MPa) with increasing the DCAP content (Figure 3D), even though the maximum value is reported for PT20 (Table 4). However, the general trend is that the progressive addition of DCAP leads to a decrease in both tensile strength and elongation, with a relative increase in Young modulus.

In the flexural test, we observe a progressive decrease in the flexural strength with increasing DCAP content (from 74.6 to ~50 MPa), in contrast with the Young modulus that shows a sawtooth pattern, with the maximum value recorded for the sample PT20 (Figure 2F and Figure 3F and Table 4).

PT samples display a general decrease in the tensile and flexural strength with increasing DCAP content, whereas the compressive strength remains constant or slightly increases. This behaviour could be explained by the weak cohesive forces between the inorganic filler and the polymer, since the particles–resin boundaries can act as cracking sites. The frequency of this phenomenon is proportional to the filler content. 

**Figure 4:**
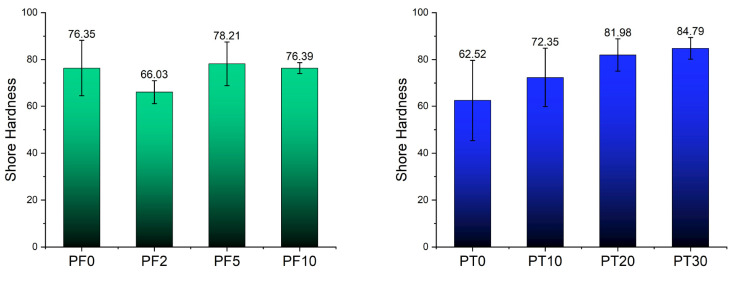
Variation of Shore hardness on PF (**left**) and PT (**right**) samples.

Figure 4 and Table 5 show the variation in the mean Shore hardness values for the PF and PT samples with increasing DCAP content. Among the PF samples, PF2 still shows the anomalous behaviour observed above, with a lower mean value (66) than the average 76–78 value of all the other samples (Figure 4). This suggests that, with the exception of PF2, the addition of DCAP in the PF resin does not substantially affect the hardness of the compound, which is probably mainly affected by pre-existing inorganic fillers (i.e., barite). PT samples, instead, show a progressive increase in the Shore hardness with increasing DCAP content, from ~62 for the PT0 sample to ~85 for the PT30 one (Figure 4). In this case, the presence of the DCAP is solely responsible for the hardness increase in the whole composite.

Although the direct comparison between the PF and PT samples is complicated by the different natures of the host polymer matrix, the marginal decrease in the main mechanical properties and, especially, the superior mechanical properties of sample PT30 over sample PF0 with similar inorganic load (Table 3, Table 4 and Table 5), suggest that DCAP can be advantageously used as a filler in substitution for commercial barite. In particular, the addition of 20 wt% of DCAP to the PT epoxy resin (PT20) confers the best mechanical properties to the composite in terms of compressive, tensile, and flexural strengths, but the addition of 30 wt% of DCAP (PT30) gives the highest Shore hardness, with only a minor worsening of all the other mechanical properties. Considering that hardness may be the most relevant property in flooring applications, being generally correlated with wear resistance, coupled with the economic advantage deriving from the addition of 30 wt% of DCAP in the more expensive polymer, the PT30 formulation may result commercially preferable.

### 3.2. Thermal Properties

**Figure 5:**
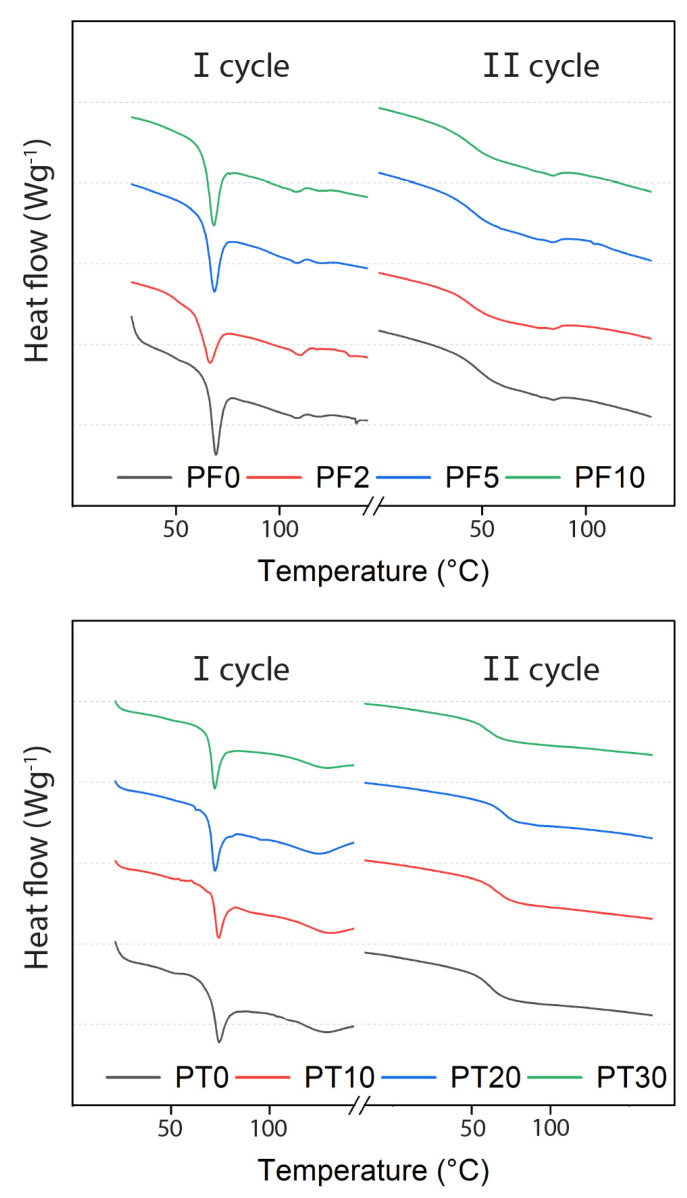
DSC data of samples PF (**upper**) and PT (**lower**).

DSC experiments were performed between −20 and 160 °C, thus starting below the conceivable utilization temperature for flooring materials and reaching a temperature above the glass transition of typical resins of this class. Until 50 °C, the thermogram does not present any feature, as expected from materials that should be stable for a long time at room temperature. Instead, endothermal events over this temperature are depicted in Figure 5 and summarized in Table 6. Regardless of the DCAP content, both PF and PT present a relevant endothermal event during the first heating. This is a typical consequence of the stress accumulated during the polymerization step that is released upon heating. The intensity of this process can vary in ways that are not directly linked to the composite composition − being strongly dependent on the detail of each single reaction, leading to an imprecise determination of the T_g_. In fact, the first heating irreversibly resets this stress, representing the thermal history of the sample. So, much more reliable results can be inferred from the second heating ramp (Figure 5), giving information about the curing of the composite. Further information that can be extracted from the first cycle relates to sample PF2. Its peak is so starkly different from those of the other samples that we cannot exclude that its specific composition is associated with some polymerization issues that also affect the mechanical properties.

Considering the second heating ramp (II cycle), PT samples consistently show a typical glass transition represented by the change in the slope for heat. Instead, PF samples show an endothermal “bathtub” path that, in addition to the Tg, indicates the formation of a mesophase. The presence of such mesophase is an indicator of microscale separation, usually found in thermoplastic elastomers, such as styrene-ethylene-butylene-styrene (SEBS) [34]. This indicates a difference between PF and PT samples, possibly due to the presence of PPG blocks that can display nanophase separation. This aspect is not directly relevant for flooring applications since it is always associated with higher temperatures than those typically experienced by resin floors but is an indicator of the diversity in terms of microenvironment of the different polymers that can host DCAP particles. In other words, it is an indication that the application of DCAP particles as fillers is not limited to a single formulation, but potentially to a wide range of polymers of actual industrial relevance.

The addition of inorganic filler may vary the T_g_ of the composite significantly, resulting in both an increase [35] or a decrease [36] depending on the nature of the filler [37]. For instance, [18] relates the change in T_g_ of the composites to the filler size, in which an increase in the interfacial area between the filler and the resin leads to a reduction in the polymer chain mobility with a subsequent increase in T_g_.

In general, we observe that the addition of DCAP in both resins does not affect the T_g_ significantly, which is ~55 °C for the PF samples and ~59 °C for PT samples. Interestingly, sample PF2 also exhibits values in line with the series. An exception is sample PT10 showing a slight increase in T_g_ at ~61.5 °C. The low dependence of T_g_ on the filler load may indicate a weak interfacial strength between the micron-sized fillers and the resin. Indeed, only a small aliquot of the DCAP (~10% by volume) has a sub-micrometric (<1 µm) size (Figure 1). This suggests that the nanosized particles are primarily responsible for directly affecting the T_g_ due to their large surface area [38]. Similar results were attained by Siddique [39] studying a low-density polyethylene polymer incorporated with reclaimed clay from oil-based mud waste.

### 3.3. Filler Dispertion and Fracture Morphology

**Figure 6:**
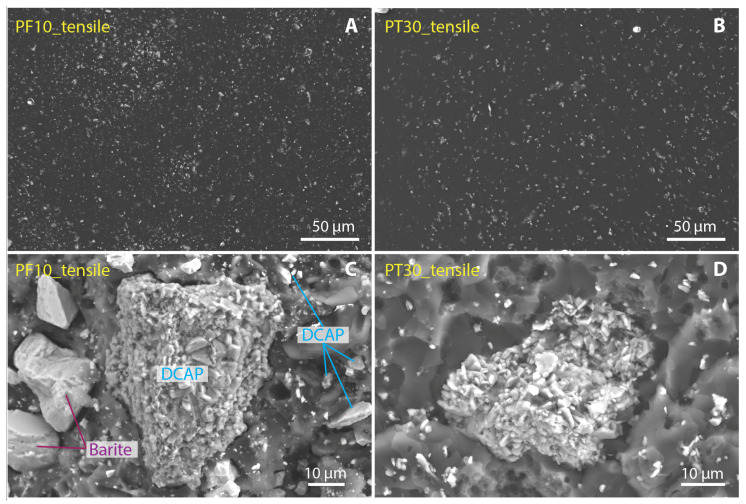
(**A**,**B**) SE images of the pristine external surface of the PF10 and PT30 samples, respectively. The bright spots are inorganic filler grains. (**C**,**D**) SE images of PF10 and PT30 samples, respectively, showing the typical shape and size of the inorganic filler. The DCAP is present either as individual micro-to-nano particles (highlighted by blue lines in (**A**)) or as aggregate up to several tens of microns in size. Barite shows a more homogeneous average grain size compared to DCAP, in agreement with DLS data (Figure 1).

SEM images of the filler dispersion in resin, the typical grain morphology, and their average particle size distribution are reported in Figure 6. Apart from some agglomerate grains in DCAP composites, the dispersion of both DCAP and barite appears to be quite homogeneous (Figure 6A,B and Figure S4 in the Supplementary Material). The proper distribution of the filler particles in the resin is a crucial aspect since any heterogeneity in the filler distribution may result in a drastic decrease in the mechanical properties of the composite. 

**Figure 7:**
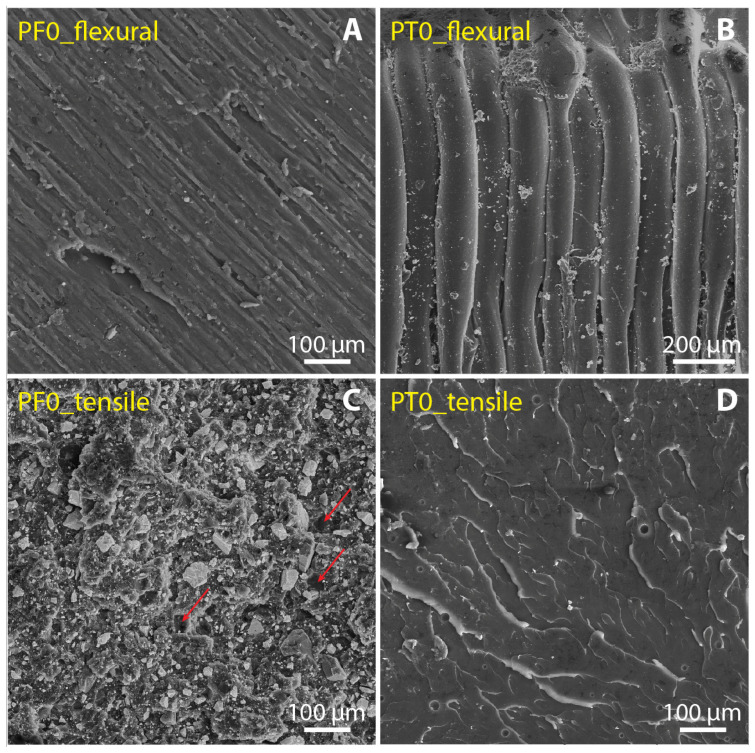
SE images of PF0 and PT0 showing the fracture surfaces after the flexural (**A**,**B**) and tensile tests (**C**,**D**).

**Figure 8:**
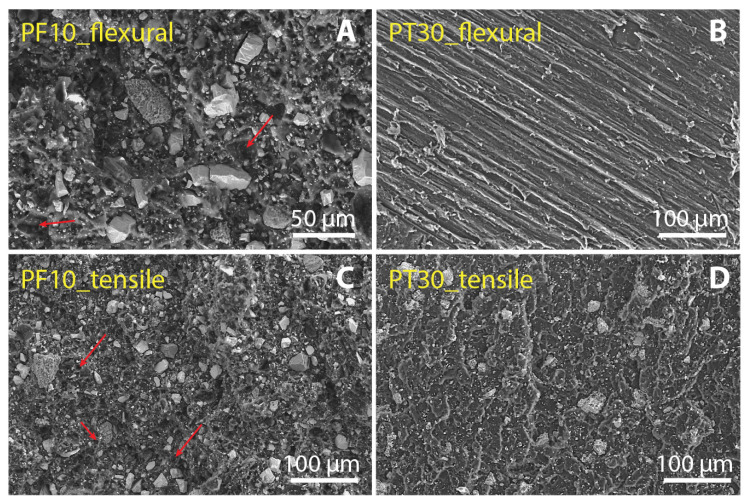
SE images of PF10 (**A**,**C**) and PT30 (**B**,**D**) samples showing the fracture surfaces after the flexural (**A**,**B**) and tensile tests (**C**,**D**).

At the SEM, the grain size distribution of the DCAP appears heterogeneous, consisting either of individual particles generally down to 1 µm in size (Figure 6A), or of aggregates with a great variability in size, up to several tens of microns (Figure 6, Figure 7C and Figure 8A,C). This is in agreement with DLS measurements (Figure 1; see also [2]), in which the DCAP shows a tri-modal distribution with a peak at ~0.8 µm (<1.5 µm: ~18% by volume), representing the individual particles seen with the SEM (for instance Figure 6B), and the peaks at ~4 (1.5–11 µm: 48% by volume) and ~40 µm (>11 µm: 34% by volume), representing the two most abundant dimensions of the DCAP aggregates observed at the SEM. 

Barite shows a slightly more homogeneous grain size distribution compared to DCAP, as is also shown by DLS (Figure 1). In fact, barite shows two major peaks, one (<1.5 µm fraction: ~17% by volume) corresponding almost perfectly with the ~0.8 µm peak of the DCAP, and one at 10–11 µm (1–80 µm fraction: 87%). A third minor peak is present at ~200 µm (>80 µm fraction: 2.6% by volume).

**Figure 9:**
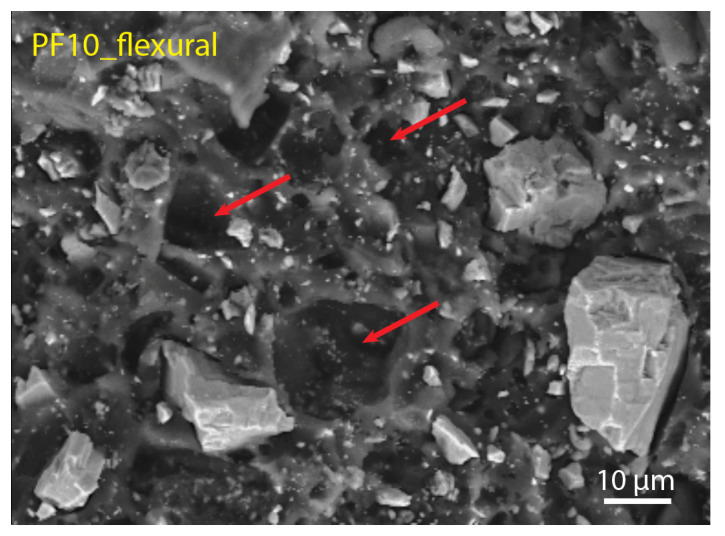
SE image showing a close-up view of the holes (highlighted by red arrows) left by the removal of the microparticles of barite due to the applied force during the flexural test on sample PF10. The same features can be seen in all PF samples (Figure 7C and Figure 8A,C), regardless of the presence of DCAP.

Regarding the fracture mechanisms, we studied the morphology of the fracture surfaces after tensile and flexural tests. Figure 7 and Figure 8 show the fracture surfaces of the reference samples (PF0 and PT0) and samples with max values of DCAP (PF10 and PT30), respectively. Contrary to PT samples, PF samples display several cavities in the resin, revealing that parts of the inorganic filler were detached during the mechanical tests. The presence of these cavities in the PF0 sample, i.e., only barite as an inorganic filler, indicates that the removed particles were barite. A close-up view of these holes is shown in Figure 9, indicated by red arrows. This suggests a poor adhesion of barite with the PF resin, in which the particle–resin boundary acted as an initial cracking site. Furthermore, these cavities are present throughout the fracture surfaces of the samples and seem to cover both the main peaks at ~0.8 µm and 10–11 µm of barite (Figure 1), indicating that the poor adhesion of barite does not depend on the particle size.

Therefore, DCAP seems to have a greater adhesion strength with resin than barite. The comparison between the gran size distributions of barite and DCAP as determined by DLS (Figure 1) shows that they have an almost identical sub-microscopic fraction (10–11% by volume), while the latter has a lower proportion of particles larger than 10 µm (~34% vs. 45% by volume). This would imply that barite has a larger mean specific area than DCAP, and therefore, assuming that other surface parameters are equal, implies a better adhesion strength of DCAP. However, as testified by SEM, the apparent larger proportion of larger grains of DCAP (35–40 µm peak) is given by its stronger agglomeration tendency, promoted by the sub-microscopic fraction, which is probably larger than it appears. These agglomerates are irregular in shape, enhancing the specific surface and favouring the adhesion strength with the resin (Figure 6). On the other hand, the larger microscopic fraction, and the higher angularity of barite grains, negatively affect their bonding strength with the resin. In fact, large particles tend to promote crack formation, due to the concentration of stress around their edges [31]. This, coupled with an inhomogeneous particle shape [32], is responsible for a more heterogeneous stress distribution and subsequent detachment of particles from the resin.

## 4. Conclusions

The mechanical and thermal properties and fracture morphology of epoxy resins filled with deactivated cement asbestos have been investigated, coming to the following conclusions:

(1) Cement–asbestos slates, a building material that still poses serious environmental and health hazards, can be detoxified by a sustainable thermal treatment, powdered and successfully mixed, potentially in large amounts, to commercial resin formulations of different compositions without any further surface treatment.
(2) The addition of DCAP filler to an epoxy resin for flooring applications that already contained barite filler (PF resin) causes a slight (but still acceptable) worsening of the main mechanical properties (compressive, tensile, and flexural strengths) with increasing DCAP content. An exception is sample PF2, showing lower elasticity and higher plasticity. Repeated tests on this formulation confirmed previous results, suggesting that 2 wt% of DCAP added to the PF resin is a critical value worthy of further investigation. The other samples instead describe a trend in mechanical properties that encourages realistic applications where loading is 10% or more.
(3) The addition of DCAP filler to the neat epoxy for flooring applications (PT resin) causes a slight decrease in the tensile and flexural strengths, with increasing DCAP content, while the compressive strength is almost unaffected, and the Shore hardness increases. Overall, the PT20 sample (20 wt% of DCAP added) shows the best technical properties.
(4) The thermal behaviour of both PF and PT samples are only marginally affected by the addition of DCAP. However, the PT samples (i.e., those bearing DCAP exclusively) show an average T_g_ of ~59 °C, which is higher than the average T_g_ (~55 °C) of the PF samples, indicating that PT is a better product for flooring applications.
(5) The comparison between PF and PT composites reveals that the main mechanical properties of the PT samples are significantly better than those of the PF0 sample, i.e., the filler-bearing sample of normal production, suggesting that DCAP can be advantageously used as a filler in substitution for commercial barite. In particular, the PT20 sample (20 wt% of DCAP filler) seems very promising for flooring applications.

## Figures and Tables

**Table 1:** Name (#) and DCAP content (wt%) of the samples studied in this work.

Pavafloor H200/E		Pavatekno Gold 200	
#	DCAP	#	DCAP
PF0	0%	PT0	0%
PF2	2%	PT10	10%
PF5	5%	PT20	20%
PF10	10%	PT30	30%

**Table 2:** Bulk chemical and mineralogical compositions of DCAP (from Vergani et al. 2021 [2]).

EDXRF Chemical Analyses				XRPD Quantitative Phase Analysis		
Compound/wt%				Phase	Chemical Formula	Abundance (wt%)
Na_2_O	0.17	CaO	47.17	Akermanite	Ca_2_Mg(Si_2_O_7_)	19.33
MgO	7.65	TiO_2_	0.23	Bredigite	Ca_13.5_Ba_0.3_Mg_1.8_Mn_0.4_Si_9_O_32_	19.25
Al_2_O_3_	3.88	MnO	0.43	Merwinite	Ca_3_Mg(SiO_4_)_2_	17.84
SiO_2_	30.28	Fe_2_O_3_	5.91	Larnite	Ca_2_SiO_4_	3.67
SO_3_	3.1	LOI	0.32	Glass		39.9
K_2_O	0.42	Sum	99.56			

LOI = loss on ignition.

**Table 3:** Results of mechanical tests (compressive, tensile, and flexural strength) for the PF samples.

	Compressive Strength			Tensile Strength			Flexural Strength	
	E_c_ (MPa)	σ_c_ (MPa)	ε (%)	E_t_ (MPa)	σ_t_ (MPa)	ε (%)	σ_f_ (MPa)	E_f_ (MPa)
**PF0**	*n* = 5			*n* = 4			*n* = 5	
**Ave.**	**839.2**	**100.9**	**48.5**	**614.1**	**21.6**	**5.2**	**39.2**	**1328.9**
St.dev.	125.5	22.1	1.6	60.9	0.7	0.2	6.0	294.1
**PF2**	*n* = 5			*n* = 5			*n* = 5	
**Ave.**	**143.4**	**112.7**	**56.8**	**256.0**	**9.2**	**33.9**	**18.0**	**484.8**
St.dev.	24.8	18.8	1.7	41.8	0.9	4.5	1.5	39.6
**PF5**	*n* = 7			*n* = 5			*n* = 5	
**Ave.**	**715.4**	**57.8**	**35.2**	**551.4**	**14.8**	**7.2**	**33.4**	**1074.0**
St.dev.	90.6	28.2	10.3	110.5	2.6	1.5	3.7	196.6
**PF10**	*n* = 5			*n* = 5			*n* = 5	
**Ave.**	**574.3**	**80.0**	**44.0**	**511.0**	**14.1**	**5.6**	**30.1**	**1004.1**
St.dev.	164.8	22.0	4.3	120.9	2.3	1.2	5.3	249.5

σ = strength; Ε = Young modulus; ε = strain/elongation; *n* = number of averaged values; Ave. = average; St.dev. = standard deviation.

**Table 4:** Results of mechanical tests (compressive, tensile, and flexural strengths) for the PT samples.

	Compressive Strength			Tensile Strength			Flexural Strength	
	E_c_ (MPa)	σ_c_ (MPa)	ε (%)	E_t_ (MPa)	σ_t_ (MPa)	ε (%)	σ_f_ (MPa)	E_f_ (MPa)
**PT0**	*n* = 5			*n* = 4			*n* = 5	
**Ave.**	**1465.3**	**67.0**	**28.0**	**774.9**	**52.6**	**5.8**	**74.6**	**2033.4**
St.dev.	160.8	4.5	19.5	182.3	4.3	0.3	8.3	338.5
**PT10**	*n* = 4			*n* = 5			*n* = 5	
**Ave.**	**1238.0**	**61.6**	**22.4**	**752.1**	**51.4**	**5.1**	**65.6**	**1813.3**
St.dev.	249.5	4.7	17.9	53.0	2.8	0.6	5.4	361.2
**PT20**	*n* = 4			*n* = 5			*n* = 4	
**Ave.**	**1488.6**	**83.6**	**38.3**	**889.7**	**42.8**	**4.0**	**63.3**	**2212.3**
St.dev.	160.9	10.9	21.7	84.7	3.5	0.4	4.1	190.5
**PT30**	*n* = 4			*n* = 5			*n* = 5	
**Ave.**	**1361.0**	**67.0**	**35.2**	**839.9**	**42.2**	**3.9**	**49.9**	**1708.7**
St.dev.	268.9	6.6	12.1	84.4	4.7	0.4	6.0	223.9

σ = strength; Ε = Young modulus; ε = strain/elongation; *n* = number of averaged values; Ave. = average; St.dev. = standard deviation.

**Table 5:** Variation of the Shore hardness on resin flooring samples with increasing DCAP content for the PF (left) and PT (right) samples.

*n* = 10	PF0	PF2	PF5	PF10	PT0	PT10	PT20	PT30
**Ave.**	**76.4**	**66.0**	**78.2**	**76.4**	**62.5**	**72.4**	**82.0**	**84.8**
St.dev.	11.9	5.0	9.3	2.3	17.2	12.5	6.9	4.6

Ave. = average; St.dev. = standard deviation; *n* = number of averaged values.

**Table 6:** Glass transition temperatures obtained with DSC for each sample.

Pavafloor H200/E		Pavatekno Gold 200	
#	T_g_ (°C), II Cycle	#	T_g_ (°C), II Cycle
PF0	58.0	PT0	58.6
PF2	54.5	PT10	61.5
PF5	55.8	PT20	59.0
PF10	55.5	PT30	58.7

## Supplementary Materials

The following supporting information can be downloaded at https://www.mdpi.com/article/10.3390/polym15061410/s1. Figure S1: TGA data of PF0; Figure S2: Raw DSC data of PF samples; Figure S3: Raw DSC data of PF samples; Figure S4: SEM images of low-load samples; Table S1: Full dataset of mechanical tests on PF samples; Table S2: Full dataset of mechanical tests on PT samples; Table S3: Full dataset of Shore hardness tests.
