# Supplementary material for: Epoxy Resins for Flooring Applications, an Optimal Host for Recycling Deactivated Cement Asbestos
Source: Polymers (Basel). 2023 Mar 12;15(6):1410. doi: 10.3390/polym15061410 (PMC10056465; doi:10.3390/polym15061410)
Supplement: Supplementary file 1 [file polymers-15-01410-s001.zip › polymers-2213032-supplementary.pdf]

Supplementary Information

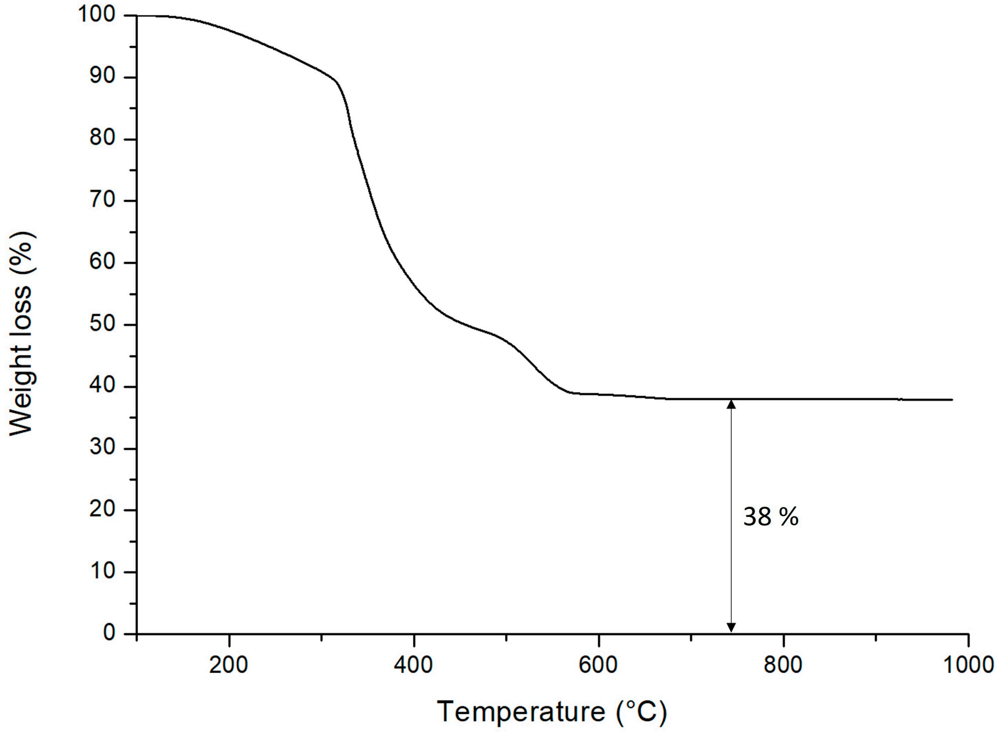

Figure SM1. TGA curve of sample PF0 revealing a content of barite of 38 wt%.

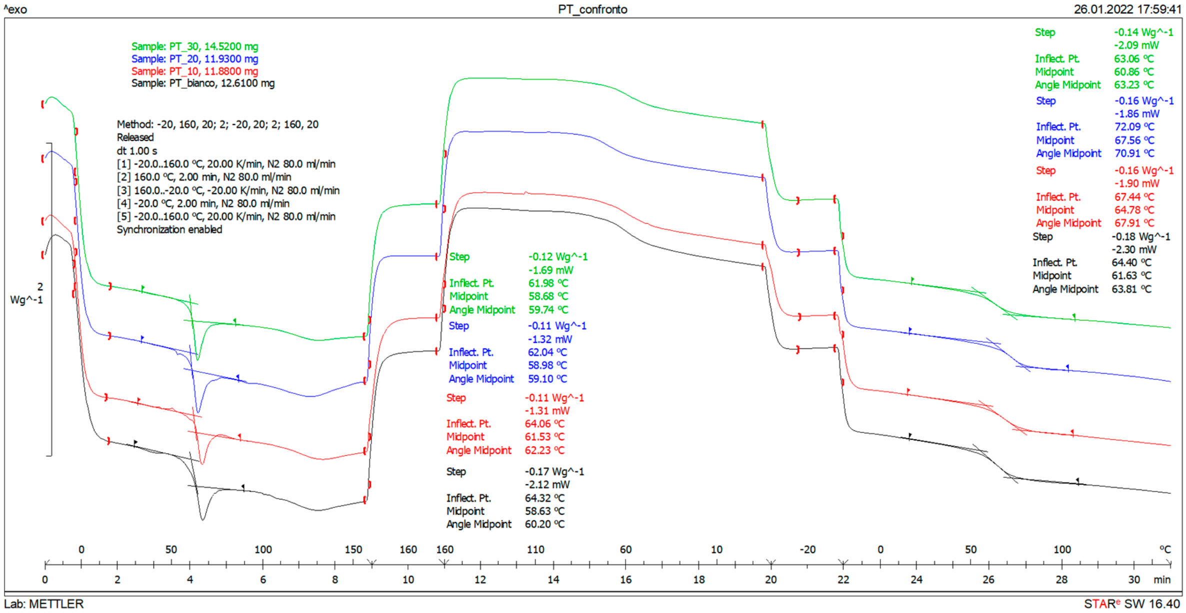

Figure SM2. DSC raw data of PT samples.

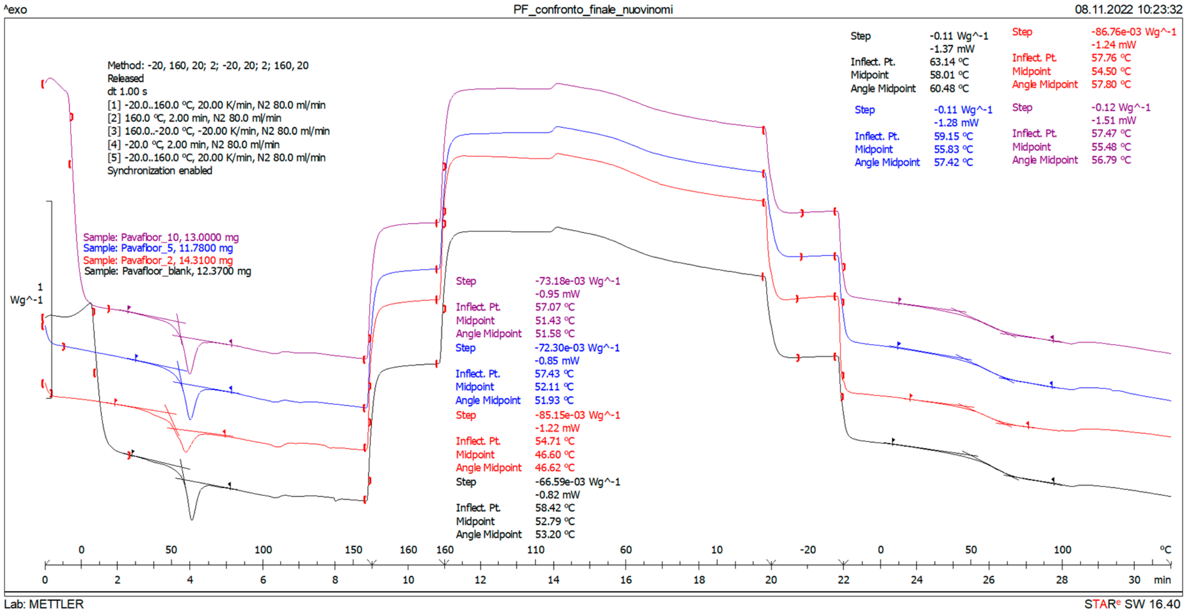

Figure SM3. DSC raw data of PF samples.

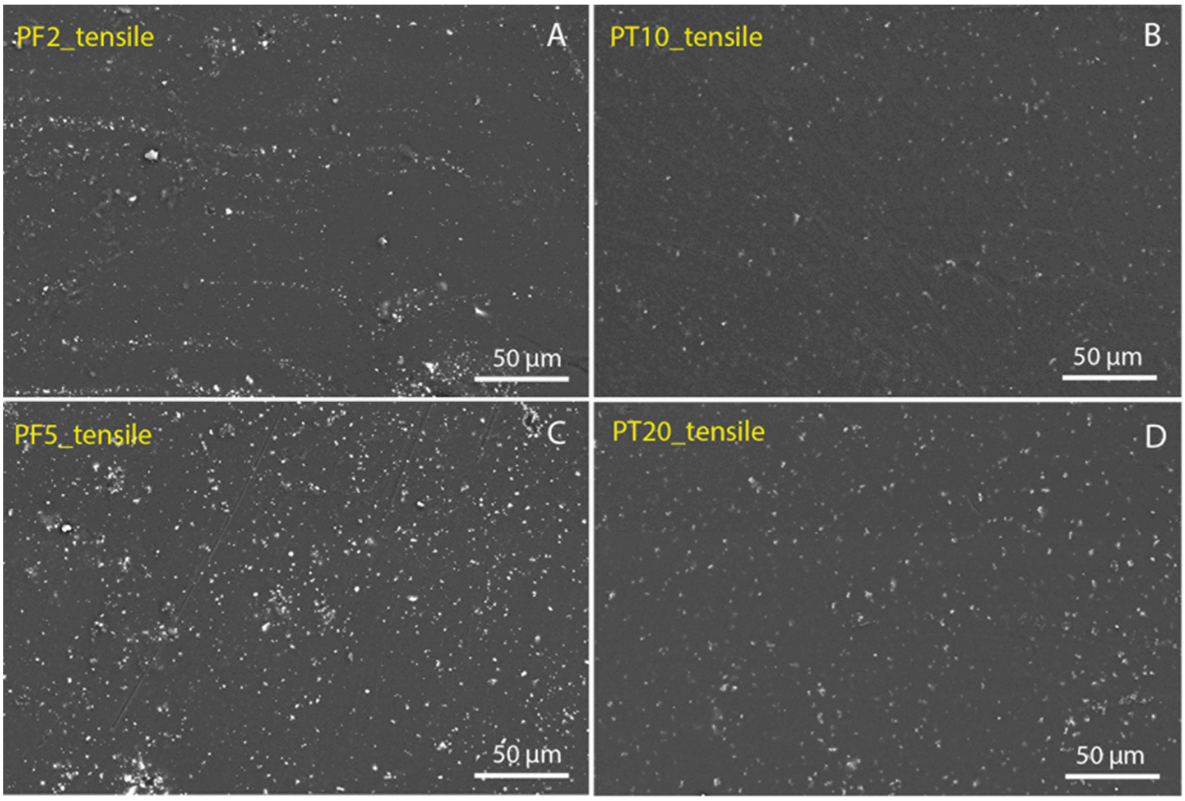

Figure SM4. SEM images showing the filler dipersion in samples PF (left) and PT (right).

Table SM1. Results of mechanical tests (compressive, tensile, and flexural strength) for the PF samples.

	Compressive strength			Tensile strength			Flexural strength	
PF0	E_c (MPa)	σ_c (MPa)	ε(%)	E_t (MPa)	σ_t (MPa)	ε(%)	σ_F (MPa)	E_f (MPa)
1	693.9	100.2	47.5	540.0	21.0	5.0	44.7	1481.6
2	870.5	132.2	51.2	597.6	22.6	5.3	38.4	1406.6
3	727.9	69.7	47.1	684.6	21.2	5.0	29.6	875.9
4	912.3	99.7	48.3	634.1	21.5	5.4	39.7	1231.8
5	991.4	102.4	48.4	-	-	-	43.8	1648.3
Ave.	839.2	100.9	48.5	614.1	21.6	5.2	39.2	1328.9
St.dev.	125.5	22.1	1.6	60.9	0.7	0.2	6.0	294.1
PF2								
1	113.2	101.2	56.5	295.6	9.6	40.2	20.3	486.6
2	167.9	129.0	59.1	282.4	10.3	28.2	18.1	440.0
3	125.4	126.6	57.4	275.3	9.2	31.3	17.2	529.4
4	168.3	85.4	54.3	232.0	9.1	35.8	18.0	517.6
5	142.2	121.2	56.8	194.5	7.9	33.9	16.3	450.2
Ave.	143.4	112.7	56.8	256.0	9.2	33.9	18.0	484.8
St.dev.	24.8	18.8	1.7	41.8	0.9	4.5	1.5	39.6
PF5								
1	794.7	45.7	34.3	493.8	12.0	8.5	37.8	1270.6
2	881.9	92.6	48.4	533.5	15.2	5.3	30.9	963.5
3	638.1	103.9	49.6	490.6	14.4	8.2	34.3	1037.2
4	667.5	48.1	33.2	492.5	13.7	8.2	35.7	1274.2
5	710.4	39.8	29.3	746.5	19.0	5.8	28.5	824.4
6	645.4	40.0	30.4	-	-	-	-	-
7	669.7	34.4	21.2	-	-	-	-	-
Ave.	715.4	57.8	35.2	551.4	14.8	7.2	33.4	1074.0
St.dev.	90.6	28.2	10.3	110.5	2.6	1.5	3.7	196.6
PF10								
1	414.2	98.2	46.2	466.2	12.1	5.8	32.6	1074.6
2	822.0	62.8	43.3	695.5	16.8	7.4	24.0	717.0
3	455.7	80.4	45.9	522.0	15.6	4.5	25.5	768.8
4	530.3	53.8	36.9	361.6	11.4	5.8	31.7	1185.8
5	649.5	105.0	47.7	509.6	14.7	4.6	36.9	1274.5
Ave.	574.3	80.0	44.0	511.0	14.1	5.6	30.1	1004.1
St.dev.	164.8	22.0	4.3	120.9	2.3	1.2	5.3	249.5

σ = strength; E = Young modulus; ε = strain/elongation; Ave. = average; St.dev. = standard deviation.

Table SM2. Results of mechanical tests (compressive, tensile, and flexural strengths) for the PT samples.

	Compressive strength			Tensile strength			Flexural strength	
PT0	E_c (MPa)	σ_c (MPa)	ε(%)	E_t (MPa)	σ_t (MPa)	ε(%)	σ_F (MPa)	E_f (MPa)
1	1443.5	70.5	42.4	575.3	50.3	6.2	84.5	2468.9
2	1437.3	69.2	42.1	672.7	47.7	5.9	73.8	2072.8
3	1728.2	71.1	42.1	882.2	56.0	5.7	62.4	1537.3
4	1430.2	62.7	6.4	969.5	56.2	5.6	72.8	2145.8
5	1287.0	61.6	6.9	-	-	-	79.7	1942.1
Ave.	1465.3	67.0	28.0	774.9	52.6	5.8	74.6	2033.4
St.dev.	160.8	4.5	19.5	182.3	4.3	0.3	8.3	338.5
PT10								
1	1553.0	62.4	5.6	730.2	50.5	5.9	62.3	1516.9
2	1312.8	65.7	38.0	772.2	51.8	5.4	70.3	2121.3
3	1097.8	54.9	8.2	693.5	47.1	4.8	71.2	2010.8
4	988.5	63.4	37.7	832.8	53.7	4.6	65.9	2081.7
5	-	-	-	731.9	54.0	4.6	58.5	1335.6
Ave.	1238.0	61.6	22.4	752.1	51.4	5.1	65.6	1813.3
St.dev.	249.5	4.7	17.9	53.0	2.8	0.6	5.4	361.2
PT20								
1	1369.1	81.8	49.2	922.7	38.5	3.7	61.5	2007.8
2	1712.6	69.4	5.9	870.6	43.1	3.8	66.7	2361.9
3	1498.7	88.5	51.8	848.3	40.5	3.5	58.4	2093.1
4	1373.9	94.8	46.2	1015.5	44.5	4.6	66.8	2386.7
5	-	-	-	791.1	47.5	4.1	-	
Ave.	1488.6	83.6	38.3	889.7	42.8	4.0	63.3	2212.3
St.dev.	160.9	10.9	21.7	84.7	3.5	0.4	4.1	190.5
PT30								
1	1244.2	73.7	48.9	738.1	40.1	4.0	51.7	1754.6
2	1061.9	58.0	31.7	965.8	36.6	3.4	47.7	1664.4
3	1685.7	68.0	39.8	857.0	48.1	4.3	43.2	1420.7
4	1452.4	68.3	20.4	843.2	40.3	3.6	59.2	2042.3
5	-	-	-	795.4	45.9	4.2	47.7	1661.3
Ave.	1361.0	67.0	35.2	839.9	42.2	3.9	49.9	1708.7
St.dev.	268.9	6.6	12.1	84.4	4.7	0.4	6.0	223.9

σ = strength; E = Young modulus; ε = strain/elongation; Ave. = average; St.dev. = standard deviation.

Table SM3. Variation of the Shore hardness on resin flooring samples with increasing DCAP content for the PF (left) and PT (right) samples.

#	PF0	PF2	PF5	PF10	PT0	PT10	PT20	PT30
1	82.0	70.3	83.3	73.9	81.7	50.0	85.8	73.7
2	83.1	57.5	66.3	75.8	72.7	87.4	83.9	82.9
3	63.9	69.1	83.9	75.7	47.3	82.0	86.2	88.6
4	87.2	67.8	80.0	77.9	85.6	78.1	85.5	87.9
5	80.8	56.8	84.6	73.4	43.9	74.7	78.8	87.6
6	78.1	67.7	75.0	78.1	44.4	58.1	81.5	87.1
7	79.5	65.2	86.3	80.5	50.4	83.9	63.6	88.1
8	77.0	66.3	58.6	73.6	69.0	80.3	85.6	83.4
9	84.2	70.8	77.5	77.2	48.7	60.5	85.3	81.7
10	47.7	68.8	86.6	77.8	81.5	68.5	83.6	86.9
Ave.	76.4	66.0	78.2	76.4	62.5	72.4	82.0	84.8
St.dev.	11.9	5.0	9.3	2.3	17.2	12.5	6.9	4.6

Ave. = average; St.dev. = standard deviation.
